# Predictors of Colorectal Resection and Primary Anastomosis outcome at Muhimbili National Hospital

**DOI:** 10.24248/eahrj.v8i2.783

**Published:** 2024-06-26

**Authors:** Jumanne Omari Masea, Fransia Arda, Godfrey Mchele

**Affiliations:** a Muhimbili University of Health and Allied Sciences, Dar es Salaam, Tanzania; b Muhimbili National Hospital, Dar es Salaam, Tanzania

## Abstract

**Background:**

Anastomotic leakage is among the most common complications after bowel resection and primary anastomosis, causing considerable morbidity and mortality. As a result it tends to affect the quality of life and increase burden to the patients and caretakers. This study focused on the assessment of the predictors and outcome of anastomotic leakage among patients who underwent large bowel surgery that involved resection and primary anastomosis.

**Methodology:**

Hospital based prospective observational study at Muhimbili National Hospital. Data of the patients who underwent colorectal resection and primary anastomosis were collected by using a structured questionnaire. Consecutive recruitment sampling technique was applied to get the required sample size and followed for 30 days. Subjects' information including age, sex, perioperative information was documented and analyzed by using Statistical Package for the Social Sciences (SPSS) version 23 software.

**Results:**

The study included 141 participants. Among those operated, 23 (16.3%) developed anastomotic leakage with a mortality rate of 30.4%. Predictors which were statistically significantly associated with anastomotic leakage were hypertension, body mass index > 30kg/m^2^, history of radiation therapy, female sex, high American Society of Anesthesiologists (ASA) grade III-IV score and peritonitis. Increased length of hospital stay, re-admission and re-operation rate together with high mortality are among the outcomes of anastomotic leakage found in this study. No loss to follow up event occurred.

**Conclusion:**

Anastomotic leakage remains a considerable problem among patients undergoing large bowel surgery at Muhimbili National Hospital. From the study site, factors such as peritonitis, HIV/AIDS, hypertension, history of radiation, obesity, high ASA score (III-IV) and female sex were found to be independent predictors of anastomotic leakage. Optimization of co-morbidities conditions before surgery, choosing best surgical option such as creating temporary stoma versus primary anastomosis in dirty wound may help to reduce the rate of anastomotic leakage.

## BACKGROUND

Anastomotic leakage is one among the major post-operative complication after bowel surgery. Following resection of any part of gastrointestinal tract (GIT) joining of the lumen is usually made in order to restore the continuity and integrity,^1,2^ Procedure for restoring this continuity called anastomosis. The incidence of anastomotic leakage (AL) globally following colorectal surgery is reported to be about 2 – 19% depending on the anastomotic site,^3–5^ with a mortality of 1.7 – 16.4%,^4,6^ While in historic studies leak rates up to 30% were reported, and reasons for decrease rates include improvement on surgical techniques and application of prevention methods.

Predictors for anastomotic leakage have been studied for a number of years, and the most identified factors mentioned are male sex (due to narrow pelvis), old age above 60 years, very proximal or very low anastomosis, malignant conditions, high American society of anesthesiologists (ASA) score, prolonged operation time, emergency surgery, preoperative chemo radiation, excessive blood loss or need of transfusion, smoking, alcohol use, co-morbidity conditions such as diabetes mellitus (DM) and hypertension, hypoalbuminemia, steroid use, post-operative non steroid anti-inflammatory drugs (NSAIDS) use, use of vasopressors to counteract hypotension and obesity.^1,5,7^

Despite advancement in patient care and modern surgical technique, anastomotic leakage still remains one of the most serious complications accounting for considerable morbidity and mortality, prolonged duration of patient's hospital stays and costs. At Muhimbili National Hospital (MNH) we also encounter the same problem of anastomotic leakage among the surgical patients undergoing bowel surgery. Assessment and identification of predictors and outcome of patients who develop anastomotic leakage at MNH will give direction to the practitioners about the trend of anastomotic leak and to have idea on which group of patients are vulnerable to develop anastomotic leakage and hence making proper decision on their best surgical option.

## METHODS

### Study Design and Setting

This was hospital based prospective observational study conducted at MNH from July 2022 to March 2023. MNH is located in Dar es Salaam Tanzania, a country with a population of over 60 million people. The hospital serves as the national referral level facility with over two thousand bed capacity. It receives both emergency and elective surgical cases from all over the country. Also the hospital serves as a teaching hospital for Muhimbili University of Health and Allied Sciences in various medical specialties. It has several admitting surgical wards and operating theatres for both minor and major surgical procedures including thoracic and gastrointestinal surgeries.

### Study Population

All patients underwent gastrointestinal surgery due to various indications at MNH from July, 2022 to March, 2023.

### Study Sample

All patients underwent resection and primary anastomosis of large bowel

### Data Collection

All patients planned for abdominal surgery from July 2022 to March 2023 due to different indications traced, identified and those who consented for surgery and participation were included in the study. Through principal investigator and two research assistants, operation lists were reviewed daily to identify patients who were planned for operation in a specific day. Theatre registry was traced and reviewed daily to capture all the operations done in a day so as not to miss and of the cases including emergency operations.

Variables including demographic data, known comorbidity conditions (diabetes mellitus, HIV/AIDS, hypertension and TB), use of alcohol and cigarette smoking were asked from the patient history. Baseline investigations which were done as a routine by the attending doctor such as hemoglobin level and albumin recorded, also weight and height measured by using weighing scale and measuring board respectively were done by researcher assistants and recorded in data collection tool. Patient's file and Jeeva electronic system was also used to collect other useful information such as proper diagnosis, indication for surgery and investigations details. All this information was collected earlier before surgery.

Post-surgery, through case notes review those who underwent large bowel resection and primary anastomosis were of interest in which further information extracted, recorded and patients were followed for 30 days. Perioperative information was documented by the surgeon and anesthetist such as indication for surgery, intra-operative findings, post-operative diagnosis, time used for surgery, any medication given intraoperatively, blood transfusion, use of vasopressors, index surgeon performed the surgery were found in recorded case notes and anesthetist form.

Further clarification from index surgeon who performed surgery or anesthetist team was requested for more information when necessary.

Anastomotic leakage group was diagnosed by the attending clinicians in the specific ward through assessment of the clinical features of the patients postoperatively. Discharge of feculent material through abdominal drain (if drain placed) or leakage of feculent contents directly from incision site was the most diagnostic modality. Other methods were imaging investigation such as computed tomography (CT) scan to look for any intra-abdominal collection for suspected patients and also, information from second look operation, because these patients they usually needed re-operation for definitive treatment. For patients who underwent second surgery a confirmation report of anastomosis breakdown or leakage was obtained from the surgeon during follow up.

Information on the leakage group, any intervention done, total hospital stays (count from the day of index surgery or from the day of re-admission if was already discharged) and the outcome (readmission, reoperation, length of hospital stay and mortality) for those who developed leakage were collected and recorded in patient structured questionnaire. Patients were followed up for 30 days post-surgery.

## METHODS

Patients' demography including age and sex were obtained from the case note by looking the year of birth and whether is male or female respectively. Comorbidities such as hypertension, diabetes mellitus, HIV/AIDS, tuberculosis were obtained from the patients' history or case notes documented by the primary attending clinician. Procedure was termed as colorectal resection and anastomosis if any part of large bowel was resected and primary anastomosis done. Preoperative laboratory findings including hemoglobin and albumin level were found in patient case note or Jeeva electronic system as the part of preoperative preparation done by attending clinician.

Indications for surgery in this study were, traumatic bowel injury defined as insult from trauma with no perforation, bowel tumor defined as presence of intestinal mass, intestinal obstruction defined as failure to pass stool or flatus not due to bowel tumor, peritonitis defined as presence of pus collection and tissue debris, bowel perforation defined as loss of bowel intergrity where by intraluminal contents communicate with external environment, enterocutaneous fistula considered if occur spontaneously not due to previous anastomosis, other indications such as diverticulitis, inflammatory bowel disease and redundant colon were taken as written by index surgeon.

Findings intra-operatively include fecal contamination defined as spillage of intestinal content in the abdominal cavity, gangrenous bowel defined as blackish discoloration of the large bowel, viable bowel defined as normal intestine, peritonitis when there was pus collection and tissue debris.

Primary outcome was occurrence of anastomotic leak within 30 days post-surgery. This was diagnosed when documented by attending physician by looking at the clinical features of leakage of feculent material per drainage tube or per incision site or when there was evidence of breakdown of the anastomotic suture line during relaparotomy. The leakage rate was calculated as the proportion of patients who developed leakage within 30 days of follow up among those who underwent colorectal surgery. Other secondary outcome of interest were readmission, reoperation, length of hospital stay and discharge status (died or alive).

### Data Analysis

Data were checked for completeness and entered into SPSS version 23 for further analysis. Categorical variable were summarized as the frequency with proportion while continuous variables were summarized into means and standard deviations

### Ethical Approval

This study was approved by Institutional Review Board of the Muhimbili University of Health and Allied Sciences (reference number MUHAS-REC-07-2022-1275) and permission to conduct the study was obtained from MNH research, education and consultancy unit (reference number MNH/TRCU/Perm/2022/107). The study did not change usual surgical practice of the facility, and there was no harm to the patients. No direct patient identifiers were used during data entry and analysis.

### Study Limitations

Limitation in this study were missing of some laboratory investigation results such as serum albumin in majority of emergency cases, therefore further studies should consider this gap in order to determine clear conclusion on the effect of albumin level in anastomotic leakage.

## RESULTS

### Socio-demographic and known co-morbidities of the study participants at Muhimbili National Hospital, n = 141

The study was conducted for period of 9 months from July 2022 to March 2023. Altogether 141 participants were included in the study, majority were male (61.7%) and age below 60 years (75.9%). Twenty-three (16.3%) developed anastomotic leakage, with female to male ratio of 2:1. Body mass index > 30kg/m2, HIV/AIDS, female sex, history of preoperative therapeutic radiation, hypertension and peritonitis were the independent predictors for anastomotic leakage in this study. Increased reoperation rate, length of hospital stay, readmission and mortality were among the secondary outcome among the leakage group (Table 1).

**Table 1.** Sociodemographic and Co-morbidities, N = 141

Variables	N	%
Age (years)		
< 60	107	75.9
60 + [Median, (IQR)]	34 [49 (25, 75)]	24.1
Sex		
Male	87	61.7
Female	54	38.3
Diabetes mellitus		
Yes	5	3.5
No	136	96.5
Hypertension		
Yes	10	7.1
No	131	92.9
Tuberculosis		
Yes	1	0.7
No	140	99.3
HIV/AIDS		
Positive	6	4.3
Negative	3	2.1
Unknown	132	93.6

### Proportion of Anastomotic Leakage Following Bowel Resection and Anastomosis, n = 141

**Figure 1.**
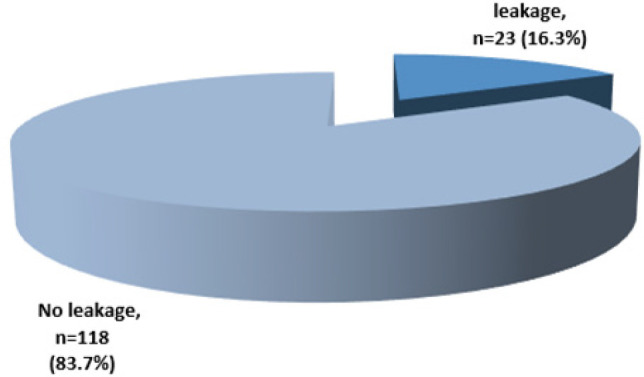
Proportion of Anastomotic Leakage Following Anastomosis at MNH, N = 141

Among 141 patients who underwent bowel resection and primary anastomosis, 23 (16.3%) developed anastomotic leakage, (Figure 1). Female were leading constituting about 14 (60.9%), male were 9 (39.1%) with female to male ratio approximately 2:1, age below 60 years were 17 (15.9%) as compared to above 60 years of age were 7 (17.6%).

### Predictors of Anastomotic Leakage

The participant characteristics and various predictors identified were tested through bivariate analysis, factors such as female sex (*P* = .015), HIV/AIDS positive (*P* = .0005), hypertension (*P* = .035), body mass index > 30kg/m^2^ (*P* = .0005), albumin level < 30g/L (*P* = .0005), history of radiation therapy (*P* = .017), ASA III-IV score (*P* = .0005) and peritonitis (*P* = .0005) were found to be statistically significantly associated with anastomotic leakage.

Other factors such as age more than 60 years, diabetes mellitus, emergency or elective surgery, hemoglobin level, duration of surgery, use of inotropes or intraoperative blood transfusion, use of protective stoma and others did not show any statistically significant association with the anastomotic leakage (Table 2).

**Table 2.** Bivariate Analysis on the Predictors of Anastomotic Leakage at Muhimbili National Hospital, N = 141

Variable	Leakage, N (%)	No leakage, N (%)	p-value
Age (years)			
< 60	17 (15.9)	90 (84.1)	
60 +	6 (17.6)	28 (82.4)	.64
Sex			
Male	9 (10.3)	78 (89.7)	
Female	14 (25.9)	40 (74.1)	.015
Diabetes mellitus			
Yes	2 (50)	2 (50)	.064
No	21 (15.3)	116 (84.7)	
HIV/AIDS			
Positive	3 (50)	3 (50)	.0005
Negative	0 (0)	3 (100)	
Not known	17 (12.9)	115 (87.1)	
Tuberculosis			
Yes	0 (0)	1 (100)	
No	23 (16.4)	117 (83.6)	.658
Hypertension			
Yes	4 (40)	6 (60)	.035
No	19 (14.5)	112 (85.50)	
Body mass index			
BMI > 30kg/m^2^	7 (70)	3 (30)	.0005
BMI < 30kg/m^2^	16 (12.2)	115 (87.8)	
Hemoglobin level			
< 10g/dl	2 (13.3)	13 (86.7)	
> 10g/dl	21 (16.7)	105 (83.3)	.741
Smoking			
Yes	1 (33.3)	2 (66.7)	.42
No	22 (15.9)	116 (84.1)	
Alcohol use			
Yes	2 (9.5)	19 (90.5)	.361
No	21 (17.5)	99 (82.5)	
Albumin level			
< 30g/L	5 (100)	0 (0)	.0005
> 30g/L	6 (8.8)	62 (91.2)	
Unknown	12 (17.6)	56 (82.4)	
History or radiation			
Yes	2 (66.7)	1 (33.3)	.017
No	21 (15.2)	117 (84.8)	
ASA classification			
ASA I-II	19 (13.9)	117 (86.1)	
ASA III-IV	4 (80)	1 (20)	.0005
Type of surgery			
Hemicolectomy	8 (13.3)	52 (86.7)	
Sigmoidectomy	1 (3.7)	26 (96.3)	
Segmental resection	12 (27.9)	31 (72.1)	.089
Total colectomy	0 (0)	1 (100)	
Low anterior resection	2 (20)	8 (80)	
Nature of anastomosis			
Ileo colic	16 (25)	48 (75)	.077
Colo colonic	3 (7.1)	39 (92.9)	
Colo rectal	4 (11.8)	30 (88.2)	
Ileo rectal	0 (0)	1 (100)	
State of the viscera and abdominal cavity			
Features of peritonitis	5 (100)	0 (0)	.0005
Fecal contamination	3 (37.5)	5 (62.5)	
Gangreneous bowel	2 (10)	18 (90)	
Non gangreneous bowel	13 (12)	95 (88)	
Duration for surgery			
> 3 hours	3 (27.3)	8 (72.7)	.3
< 3 hours	20 (15.4)	110 (84.6)	
Intraoperative blood transfusion			
Yes	2 (22.2)	7 (77.8)	.62
No	21 (15.9)	111 (84.1)	
Intraoperative use of inotropes			
Yes	5 (27.8)	13 (72.2)	.159
No	18 (14.6)	105 (85.4)	
Nature of the surgery			
Emergence	12 (19.7)	49 (80.3)	.346
Elective	11 (13.8)	69 (86.2)	
Application of protecting stoma			
Yes	1 (14.3)	6 (85.7)	
No	22 (16.4)	112 (83.6)	.882

### Multivariate Logistic Regression Analysis on the Predictors Of Anastomotic Leakage at Muhimbili National Hospital, n = 141

Variables with *P* < .05 from bivariate analysis were run into multivariate logistic regression analysis to control for the confounders. In this study factors that remained significantly associated with anastomotic leakage were peritonitis (*P* = .0005), HIV/AIDS (*P* = .0005), hypertension (*P* = .036), body mass index > 30kg/m2 (*P* = .0005), female sex (*P* = .015), radiation therapy (*P* = .017) and high ASA score (*P* = .0005) (Table 3).

**Table 3.** Multivariate Logistic Regression Analysis on the Predictors of Anastomotic Leakage at Muhimbili National Hospital, N = 141

Variable	Bivariate analysis		Multivariate analysis AOR (95% CI)	p - value
	OR (95% CI)	p-value		
Peritonitis	24.6 (2.6 - 232.4)	.0005	1 (1.8-1.9)	.0005
HIV/AIDS	6.7 (1.3-36)	.026	4.7 (2.7-2.9)	.0005
History of radiation therapy	11 (0.9 - 128)	.017	1.1 (1.91-1.98)	.046
High ASA score (III &IV)	1.4 (0.04-0.3)	.005	1.6 (1-1.13)	.0005
Hypertension	3.9 (1-15)	.035	1.4 (1.83-1.95)	.036
Serum albumin level	8 (2-114)	.0005	0.6 (2.2-2.5)	.371
Female sex	3.6 (2-13)	.015	2.1 (1.9-3)	.015
Body mass index > 30kg/m^2^	16.7 (3.9-71)	.0005	1.7 (1.7-1.9)	.0005

## DISCUSSION

Despite advancement in patient care and modern surgical technique, anastomotic leakage still remains one of the most serious complications accounting for considerable morbidity and mortality, prolonged duration of patient's hospital stays and high healthcare costs. Here at MNH we also encounter this problem of anastomotic leakage among the surgical patients.

### Socio-Demographic Characteristics

In this study a total of 141 participants were included, majority were male about 61.7%, median age of 49 years with a range of 18 to 89 years, 75.9% were below 60 years of age. Similar findings in demographic data distribution have been reported in other studies conducted in Tanzania and Ethiopia.^7,8^ Similarity maybe attributed to the same geographical location as dominated by people with related disease conditions and co-morbidities.

### Incidence of Anastomotic Leakage

Globally the incidence of anastomotic leakage ranges from 2 - 19%.^3^ Variations have been observed in different regions whereby reported incidences were 8.4% in China^4^, 3% in the United State of America at the university of Michigan^3^, and 8.7% in a study done in Egypt.^6^ In Ethiopia two studies reported incidences of 5.2% and 10.8%.^8,9^ In this study the proportion of anastomotic leakage following colorectal resection and primary anastomosis was found to be 16.3%, which is higher as compared findings from other African countries and globally. This observation could be due to poor decision making on when to anastomose primarily versus creating a stoma in dirty or infected peritoneal environment, however the rate is lower as compared with the study conducted in Tanzania at Iringa regional referral hospital in 2020 revealed incidence of 19%,^7^ however no clear reason for this slight increase in incidence at Iringa Regional Referral Hospital.

### Predictors of Anastomotic Leakage Following Anastomosis

In this study, factors such as hypertension (*P* = .035), HIV/AIDS (*P* = .0005), peritonitis (*P* = .0005), high ASA (III-IV) score (*P* = .0005), female sex (*P* = .015), history of radiation therapy (*P* = .017) and body mass index of more than 30kg/m^2^ (*P* = .0005) were the independent predictors of anastomotic leakage. These findings are similar to some other studies done in China revealed body mass index more than 25kg/m^2^ and preoperative radiotherapy (decreased microvessel density hence impairment of blood supply at anastomotic site) were independent factor for anastomotic leakage.^10,11^ Hypertension and hypoalbuminemia were the independent factors for leakage in a study conducted in Egypt.^6^ Other relevant studies from Ethiopia and China revealed high ASA score > 3 as independent predictor for leakage.^8,10,11^ HIV/AIDS, peritonitis were seen as independent risk factor for leakage in a study conducted in Tanzania at Iringa regional referral hospital.^7^

### Outcomes of Anastomotic Leakage

Increased rate of relaparotomy (86.9%) among leakage group as compared to 1.7% in non leakage group was noted in this study as compared to the study done at Iringa RRH which revealed relaparotomy rate of 84.6%.^7^ Increased number of re-admission, prolonged length of hospital stay 8-44 days compared to 3-37 days in non leakage (*P* = .0005), Intensive Care Unit (ICU) admissions (17.4%) compared to none in non leakage patients were among the outcomes found. Similar findings were observed in other studies.^12–18^

Mortality rate from this context was higher (30.4%) as compared to study done at Iringa regional referral hospital reported the rate of 26.7%, another study in Spain reported mortality of 13.4% in leakage group as compared to 2.3% in non leakage group.^19^ In Sweden one study reveals mortality of 3.9% in those with anastomotic leakage as compared to 1.5% in those patients with no leakage.^20^ However the higher mortality reported in one study conducted in Ethiopia in 2019 with a rate of 50% mortality.^8^ High mortality rate among African countries maybe attributed by limited resources for provision of better health service.

## CONCLUSION

From this study, factors such as HIV/AIDS, hypertension, history of previous radiation therapy, obesity, high ASA score, female sex and peritonitis were found to be independent predictors for anastomotic leakage.

The outcomes were increased length of hospital stay, re-admission, reoperation and high mortality. All these lead to increase in treatment cost and burden to the caretakers and health service providers. Therefore, measures to reduce the rate of anastomotic leakage and its complications are of paramount importance for the better of our clients.

### Recommendation

Strategies to reduce the rate of anastomotic leakage and its outcome. Optimizing co-morbidities (Hypertension and hypoalbuminemia) before major bowel surgery; Proper decision on best surgical option especially in emergence setting, such as creation of a stoma instead of making primary anastomosis in dirty abdominal environment.
